# Supplementary material for: Fast three-color single-molecule FRET using statistical inference
Source: Nat Commun. 2020 Jul 3;11:3336. doi: 10.1038/s41467-020-17149-w (PMC7335206; doi:10.1038/s41467-020-17149-w)
Supplement: Supplementary file 1 — Supplementary Information [file 41467_2020_17149_MOESM1_ESM.pdf]

Supplementary Information

Fast three-color single-molecule FRET using statistical inference

Yoo *et al*.

This PDF file includes:

Supplementary Figures 1 to 11
Supplementary Tables 1 to 4

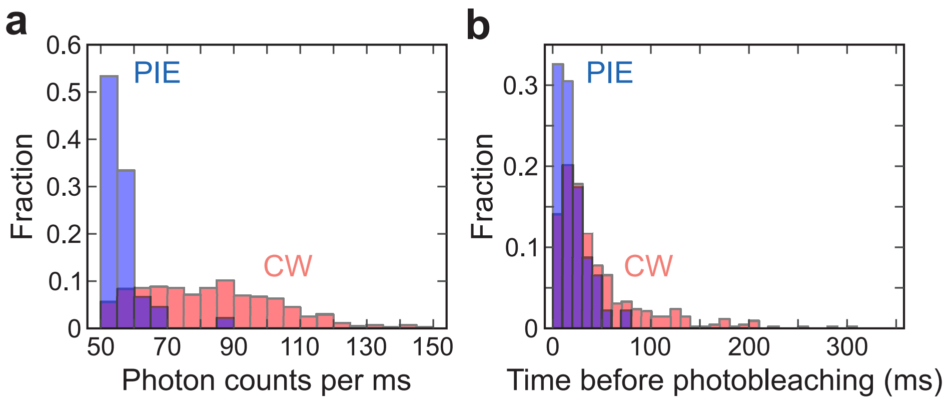

Supplementary Fig. 1. Comparison of the photophysical properties in the three-color experiments of α3D folding with alternating excitation with picosecond pulsed lasers (ALEX) and continuous wave (CW) excitation. **a** Average photon count rates of individual segments. The mean values of the distributions are 56.3 and 82.6 ms^{-1} for PIE and CW, respectively. **b** The length of three-color segments before photobleaching. The mean values of the distributions are 19.3 and 45.0 ms for PIE and CW, respectively.

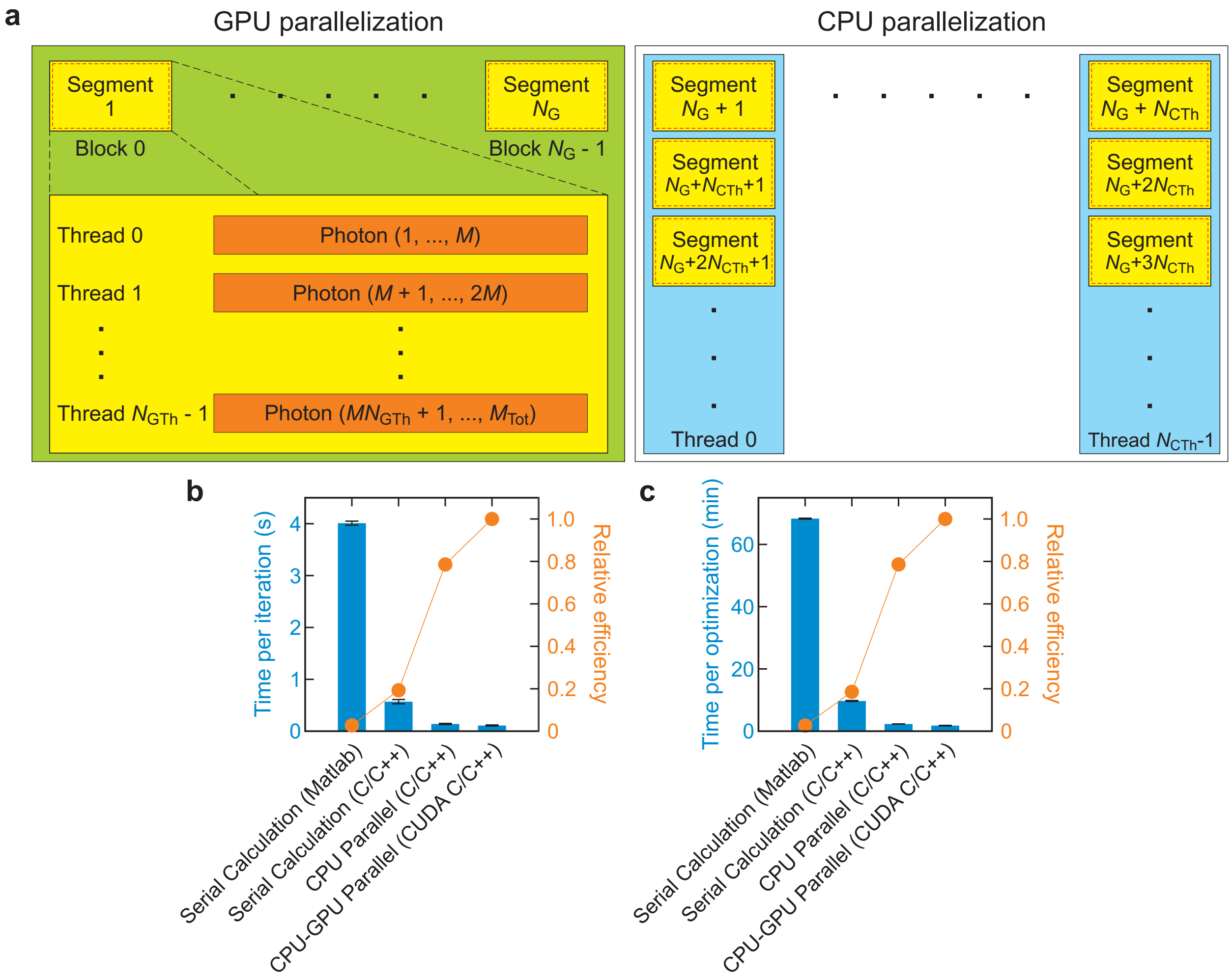

Supplementary Fig. 2. CPU-GPU co-parallelization for the likelihood calculation. **a** In the GPU parallelization, the calculation of the likelihood function of an independent segment of a photon trajectory is assigned to one of N_G GPU blocks consisting of N_{GTh} threads. Each thread (except the last one) performs matrix multiplications for M photons (see equations (28) and (29) in Methods). M_{Tot} is the total number of photons in a segment. In the CPU parallelization, segments are distributed among N_{CTh} CPU threads. In each thread, the likelihood values of the assigned segments are calculated sequentially using equation (10). **b, c** Comparison of the parallelization efficiency of the likelihood evaluation for the α_3D folding experimental data (2 M GdmCl). Source data are provided as a Source Data file. **b** The average time to calculate the likelihood function once (one iteration). **c** The average optimization (parameter determination) time. The relative efficiency (right axis) is the inverse of the time normalized to the value of the CPU-GPU parallelization. Analyses were performed for 40 3-color segments, 84 DA1 segments and 107 DA2 segments (701,980 photons) with Intel® Xeon® CPU E5-2620 v3, NVIDIA Quadro M2000, MATLAB 2018b, Visual Studio 2015 and CUDA 8.0 on Windows 10. Errors are standard deviations of 10 benchmark tests.

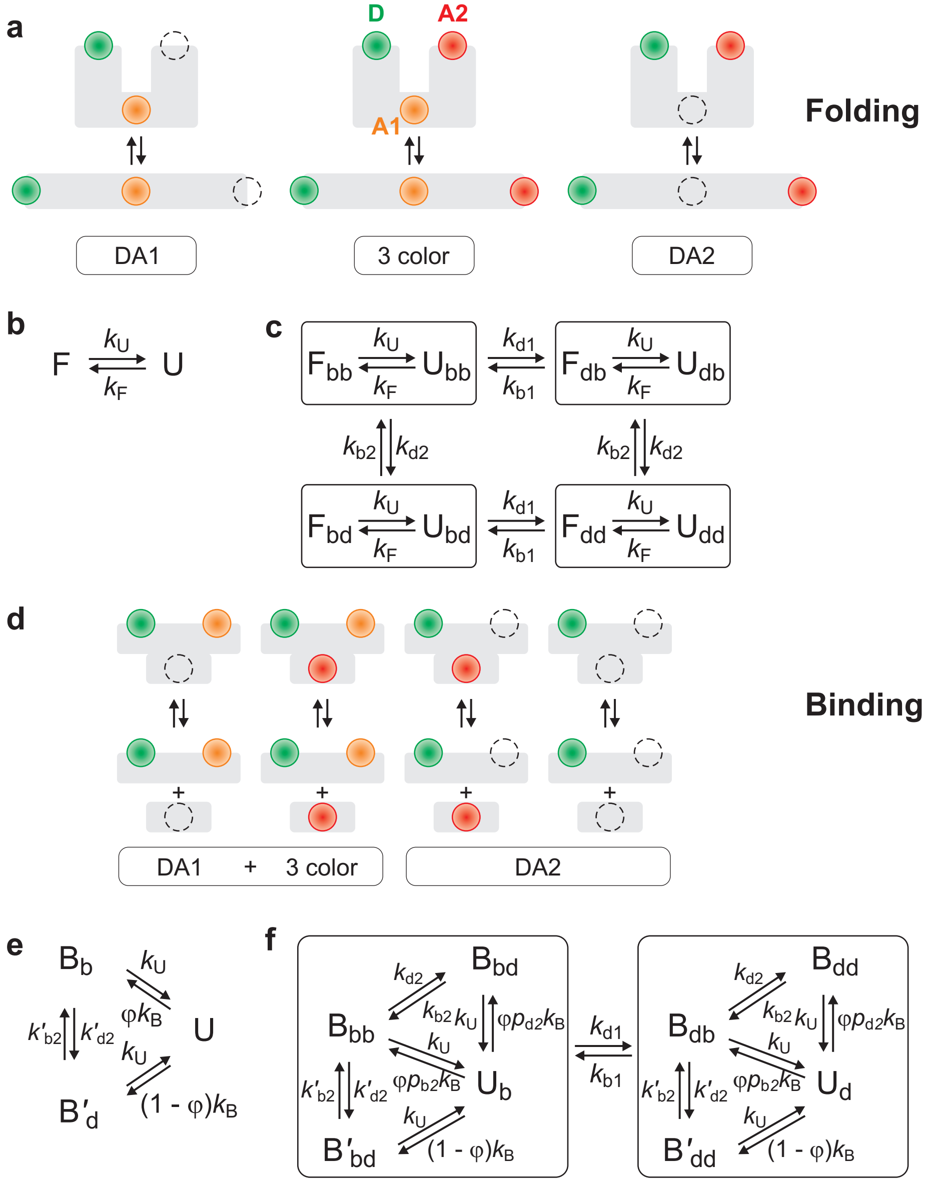

Supplementary Fig. 3. Two-state folding and binding in three-color FRET and kinetic models. **a** In the folding experiment, a protein (light gray) switches between the folded and unfolded states. Three-color (with three active labels), DA1 (A2 inactive, dashed), and DA2 (A1 inactive) photon segments can be analyzed independently. **b** Two-state model for α3D folding. F and U stand for the folded and unfolded states. **c** Two-state model with acceptor blinking (eight-state model) for α3D folding. Subscripts b and d stand for bright and dark states of acceptor 1 (first subscript) and acceptor 2 (second subscript), respectively. $k_{d(b)1}$ and $k_{d(b)2}$ are the rates from the dark (bright) to the bright (dark) states of acceptor 1 and 2, respectively. The folded and unfolded states with different acceptor photophysical states are divided into different blocks. Blinking transitions occur between the folded states and between the unfolded states in different blocks. **d** In the binding experiment, D- and A1-labeled protein can bind both A2-labeled and A2-unlabeled (inactive) binding partners. Therefore, photon trajectory segments with three-color emission and D and A1 emission (DA1) must be analyzed together. Proteins are shown in light gray. The plus sign means that the binding partners are unbound and far apart. **e** Model for two-

state binding of TAD and NCBD without acceptor blinking. U is unbound state and B_b and B'_d are bound (and folded) states with active and inactive acceptor 2, respectively. φ is the labeling efficiency of acceptor 2 (i.e., fraction of active A2). **f** Model for two-state binding of TAD and NCBD including acceptor blinking. B_{bd} is the bound state with acceptor 1 bright and acceptor 2 dark state during fast acceptor 2 blinking, which has the same parameters of acceptor fractions (ε's) as B'_{bd}, the bound state without active acceptor 2. States with different acceptor 1 photophysical states are separated into two blocks. Note that acceptor 1 blinking occurs only between the pairs with different acceptor 1 states (e.g., B_{bd} and B_{dd}) of the two blocks.

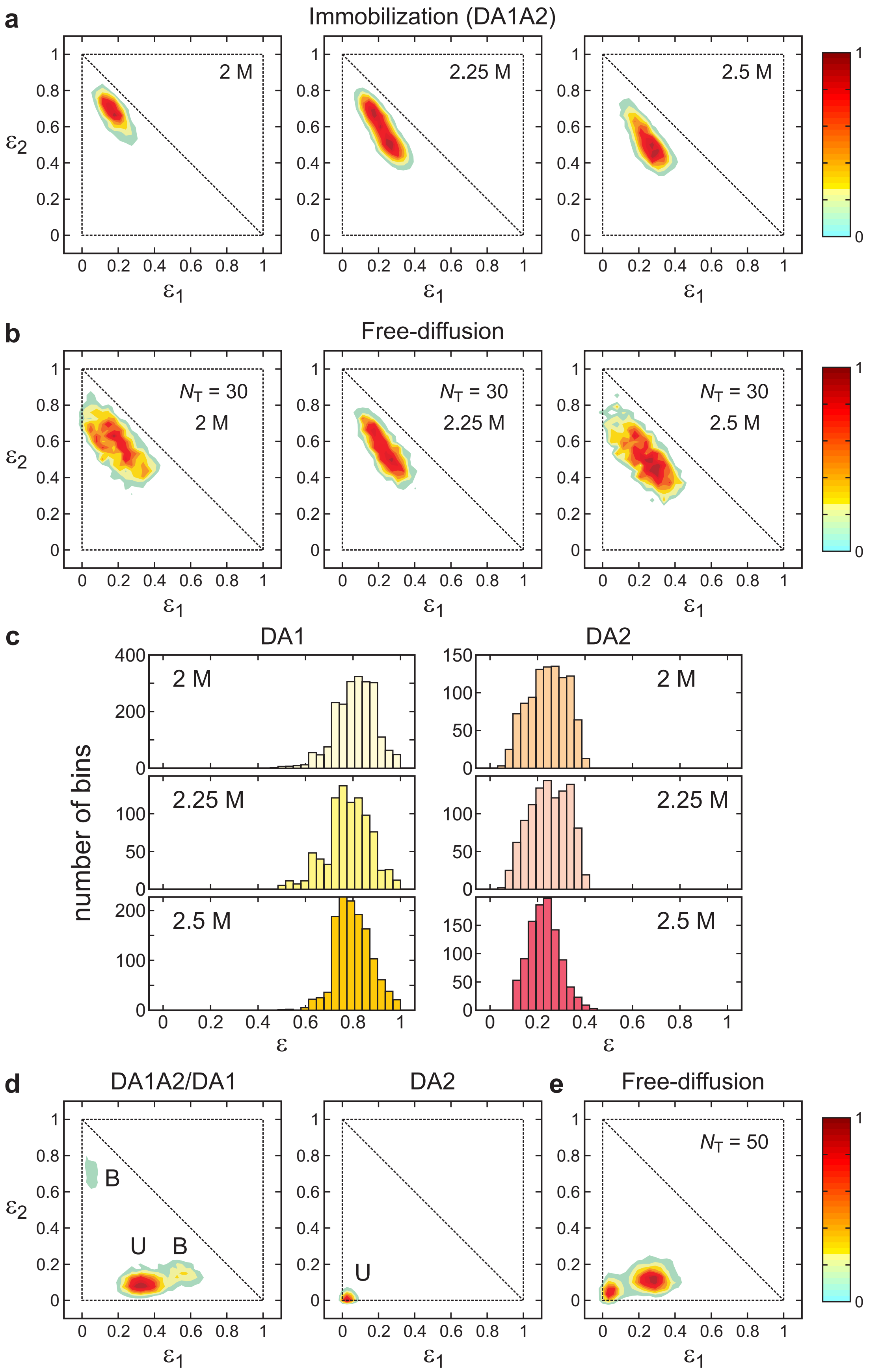
a
Immobilization (DA1A2)
2 M
2.25 M
2.5 M
ε2
ε1
b
Free-diffusion
NT = 30
2 M
2.25 M
2.5 M
c
DA1
DA2
number of bins
2 M
2.25 M
2.5 M
ε
d
DA1A2/DA1
DA2
B
U
e
Free-diffusion
NT = 50

Supplementary Fig. 4. Comparison of one- and two-dimensional histograms of acceptor fractions (ε_2 vs. ε_1) from immobilization and free-diffusion experiments. **a - c** α_3D folding (2, 2.25, and 2,5 M GdmCl). **a** Acceptor fractions were calculated from the three-color DA1A2 trajectories collected from the immobilization experiment. One-dimensional histograms of ε_1 and ε_2 are shown in Fig. 3b. **b** Acceptor fractions were calculated from the fluorescence bursts with 30 photons and more (N_T) in the free-diffusion experiment (2 ms bin time). 1707, 1444, and 1757 bursts were analyzed for the data at 2, 2.25, and 2.5 M GdmCl, respectively. Bursts of molecules containing all three active fluorophores (DA1A2) were determined based on acceptor fractions, which are easily separable from DA1 and DA2 bursts in **c**. The location and shape of the distribution are similar to those from the immobilization experiment in **a**. **c** One-dimensional histograms of acceptor fractions for DA1 and DA2 bursts. The distributions are similar to those from the immobilization experiment shown in Fig. 3c, but the width is broader due to the larger shot noise. 2129 (2 M), 886 (2.25 M), and 1303 (2.5 M) DA1 bursts, and 999 (2 M), 1060 (2.25 M), and 992 (2.5 M) DA2 bursts were analyzed. **d, e** TAD/NCBD binding. **d** Acceptor fractions were calculated from the fluorescence trajectories collected from the immobilization experiment at 0 mM NaCl (25 nM NCBD). U is the unbound state. B's on the upper left side and lower right side indicate the bound state with active A2 and inactive A2, respectively. **e** Acceptor fractions were calculated from the fluorescence bursts with 50 photons and more in the free-diffusion experiment (30 mM NCBD and 0 mM NaCl, 2 ms bin time). 3454 bursts were analyzed. Unlike immobilization experiment, separation of the bursts by molecules containing three fluorophores (DA1A2) from those containing two fluorophores (DA1 or DA2) are difficult because of the broader width of the histogram due to larger shot noise. In addition, due to the low γ-factor of A2 (γ_2 = 0.49 for TAD/NCBD binding), bursts from the bound state are effectively suppressed with the photon count below the threshold (N_T = 50) and the bound state peaks are not observed in the plot.

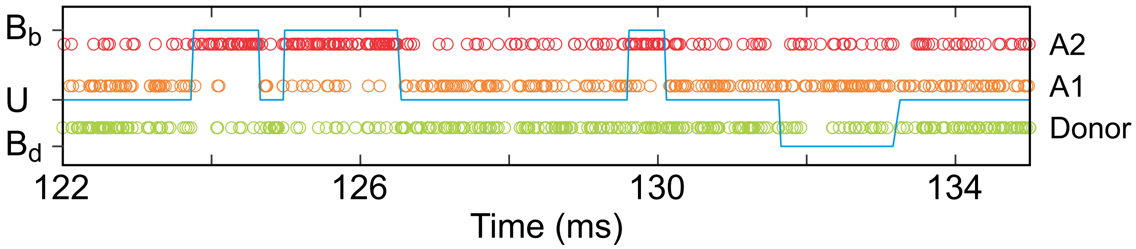

Supplementary Fig. 5. Identifying binding and dissociation transitions of TAD and NCBD at the single photon level using the Viterbi algorithm. The trajectory was collected at 30 mM NaCl. Blue solid line indicates transitions and different molecular states. In the bound state with active A2 (B_b), A2 photon count rate is the highest due to the energy transfer. In the bound state with inactive (or absent) A2 (B_d), A1 count rate is the highest due to the transfer from D to A1 and lack of transfer to A2. In the unbound state (U), the donor count rate is the highest because of the relatively low energy transfer rate from D to A1.

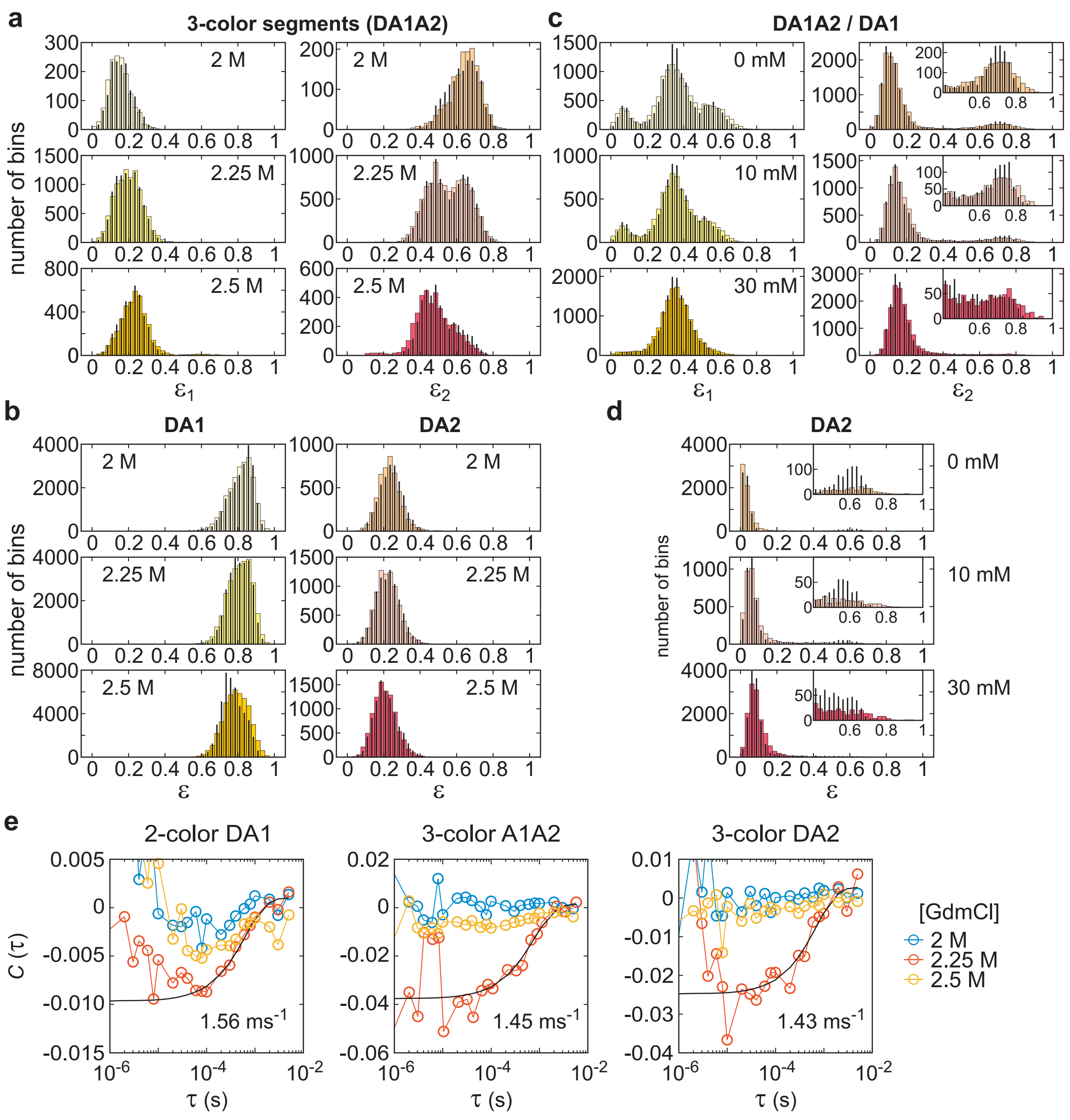

Supplementary Fig. 6. Validation of the maximum likelihood parameters. **a - d** Comparion of experimental histograms with histograms constructed from recolored photon trajectories using the extracted parameters. Simulated histograms (narrow black bars) are overlaid with experimental histograms (wide bars). **a, b** Acceptor photon fraction histograms of α3D at different GdmCl concentrations (2, 2,25 and 2.5 M) for the fractions of A1 (ε_1) and A2 (ε_2) from three-color segments (DA1A2) (**a**) and for the fractions of acceptor photons (ε) from two-color segments (DA1 and DA2) (**b**). **c, d** Acceptor photon fraction histograms of TAD-NCBD binding at different NaCl concentrations (0, 10, and 30 mM) for the fractions of A1 (ε_1) and A2 (ε_2) from three-color (DA1A2)/DA1 segments (**c**) and for the fractions of acceptor photons from DA2 segments (**d**). Enlarged histograms of the bound state ($0.4 < \varepsilon_2 < 1$) are shown in the insets.

The histograms constructed from the recolored photon trajectories using the extracted parameters (8-state model) agree well with the experimental histograms, except overestimation of the bound state peak of the DA2 histograms in (**d**). This discrepancy may result from the inaccuracy of the determination of the slow blinking rates of A2 (k'_{b2} and k'_{d2} , see Supplementary Table 2) and overall very low fraction of the bound state peak. **e** Cross-correlation analysis of α_3D folding. Cross-correlation functions were calculated for D and A1 for two-color DA1 segments (left), A1 and A2 for three-color DA1A2 segments (middle), and D and A2 for three-color DA1A2 segments (right). Due to the small differences between acceptor fraction values of the folded and unfolded states, the amplitude of correlation function is generally small. Therefore, only the data at 2.25 M, where the amplitude is the largest due to the similar folded and unfolded populations, were fitted to a single exponential function, which results in the relaxation rates of 1.56 (± 0.20) ms^{-1} (left), 1.45 (± 0.16) ms^{-1} (middle), and 1.43 (± 0.36) ms^{-1} (right).

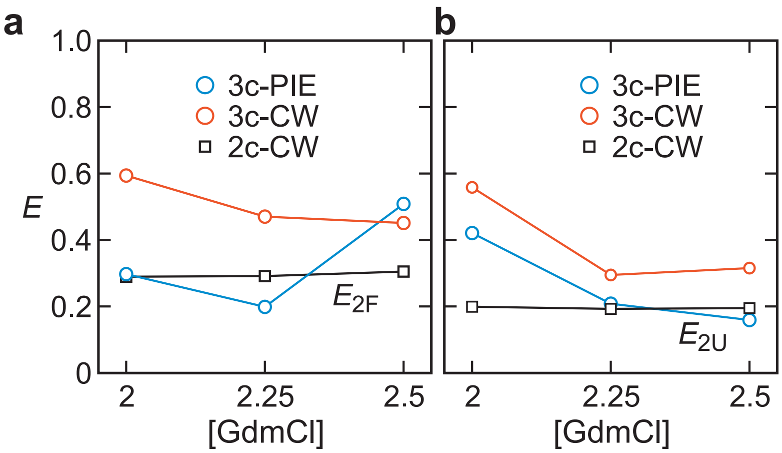

Supplementary Fig. 7. Comparison of the FRET efficiency E_2^{3c} of α3D folding in 3c-PIE and 3c-CW determined using two-color E_1. **a** Folded state FRET efficiency E_{2F} from 2 color (black), 3c-PIE (blue) and 3c-CW (red). **b** Unfolded state FRET efficiency E_{2U} from 2 color (black), 3c-PIE (blue) and 3c-CW (red). E_2^{3c} values of 3c-CW were determined using E_1^{2c} from two-color (DA1) segments. E_2^{3c} values of 3c-PIE were calculated using E_{12}^{2c} obtained from A1 excitation. Source data are provided as a Source Data file.

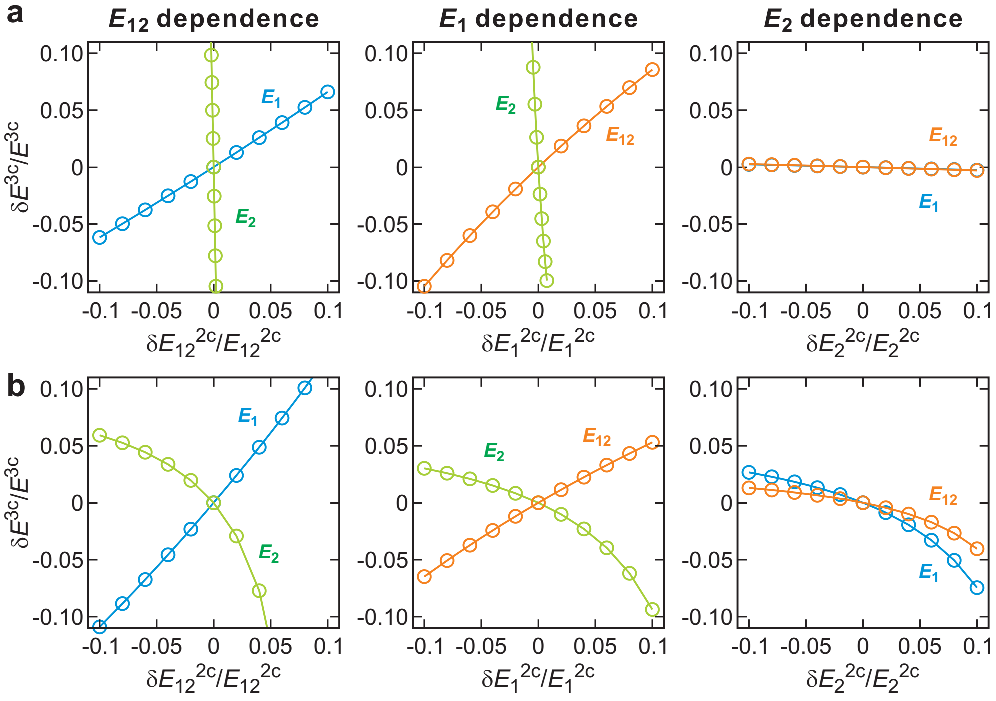

Supplementary Fig. 8. Propagation of errors from the two-color FRET efficiency to the determination of three-color FRET efficiencies calculated using equations (6) – (8). Two-color values E_{12} (left), E_1 (middle), and E_2 (right) are used to determine three-color FRET efficiencies. **a** α_3D folding. **b** TAD/NCBD binding. Source data are provided as a Source Data file.

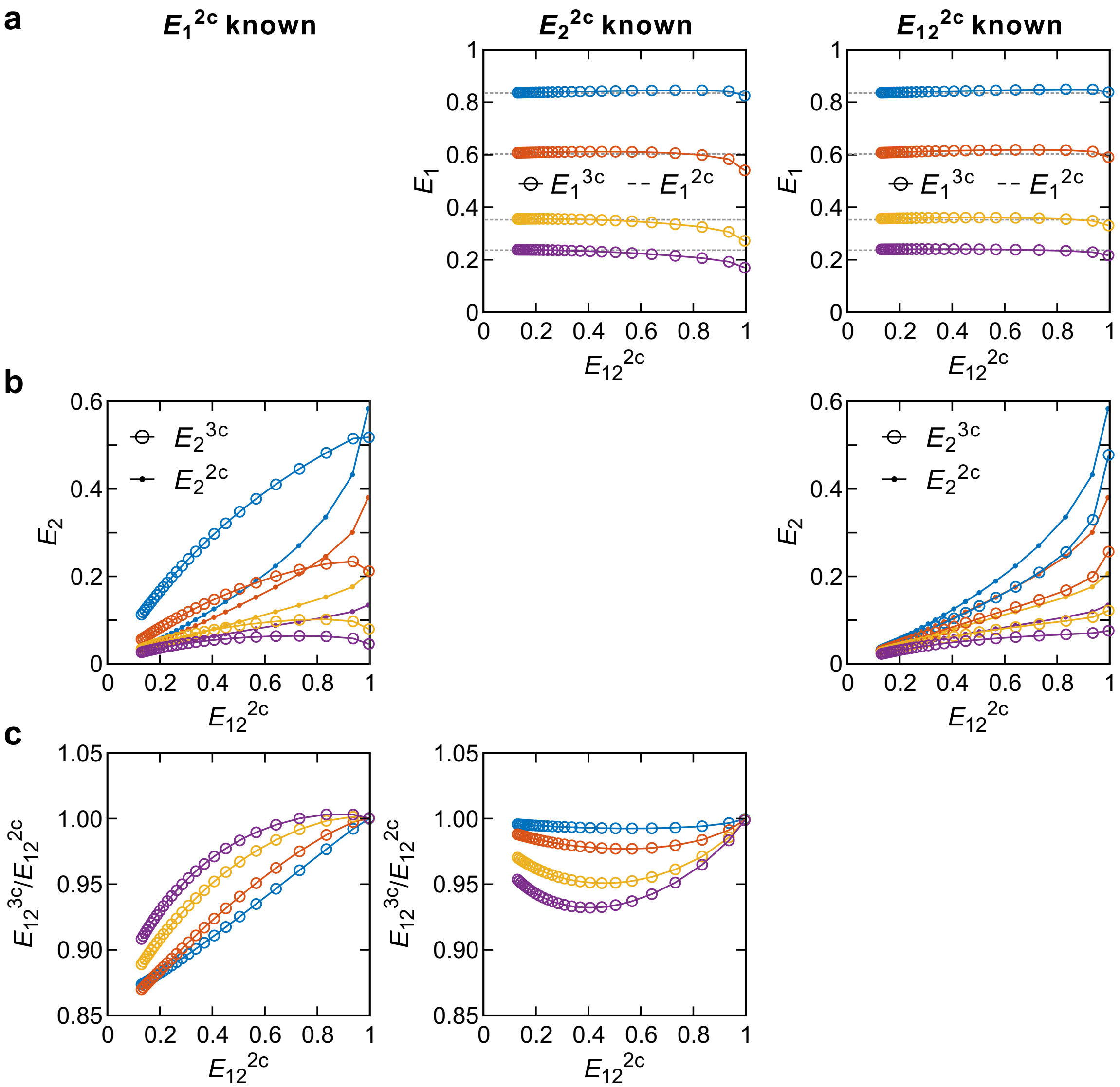

Supplementary Fig. 9. Difference between the two-color FRET efficiencies and the three-color FRET efficiencies calculated using the Gaussian chain model for the unfolded state protein. Three-color FRET efficiencies were calculated for the cases that E_1 is known (left) using equation (7), E_2 is known (middle) using equation (8), and E_{12} is known (right) using equation (6), respectively, with acceptor fractions $\varepsilon_i = \langle n_{Ai} \rangle / (\langle n_{A1} \rangle + \langle n_{A2} \rangle + \langle n_D \rangle)$ (i = 1, 2) and the average photon count rates $\langle n_{A1} \rangle$, $\langle n_{A2} \rangle$, and $\langle n_D \rangle$ calculated using equations (25) – (27). Two-color FRET efficiencies were calculated using equation (24). The parameters of the Gaussian model in equation (23), $\langle r_1^2 \rangle$ and $\langle r_{12}^2 \rangle$, were varied resulting in a set of E_{12}^{2c} and four different E_1^{2c} values: 0.834 ($\langle r_1^2 \rangle = 0.5R_1^2$, blue), 0.603 ($\langle r_1^2 \rangle = R_1^2$, red), 0.352 ($\langle r_1^2 \rangle = 2R_1^2$, yellow), and 0.236 ($\langle r_1^2 \rangle = 3R_1^2$, purple). The Förster radii, R_1 = 5.4 nm, R_2 = 4.3 nm and R_{12} = 7.0 nm were used in the calculation. **a** Comparison of calculated three-color, E_1^{3c}, (circle) and two-color, E_1^{2c}, (horizontal dashed lines) FRET efficiencies. **b** Comparison of three-color, E_2^{3c}, (circle) and two-color, E_2^{2c} (dot) FRET efficiencies. **c** The ratio of the three-color and two-color E_{12} values. Source data are provided as a Source Data file.

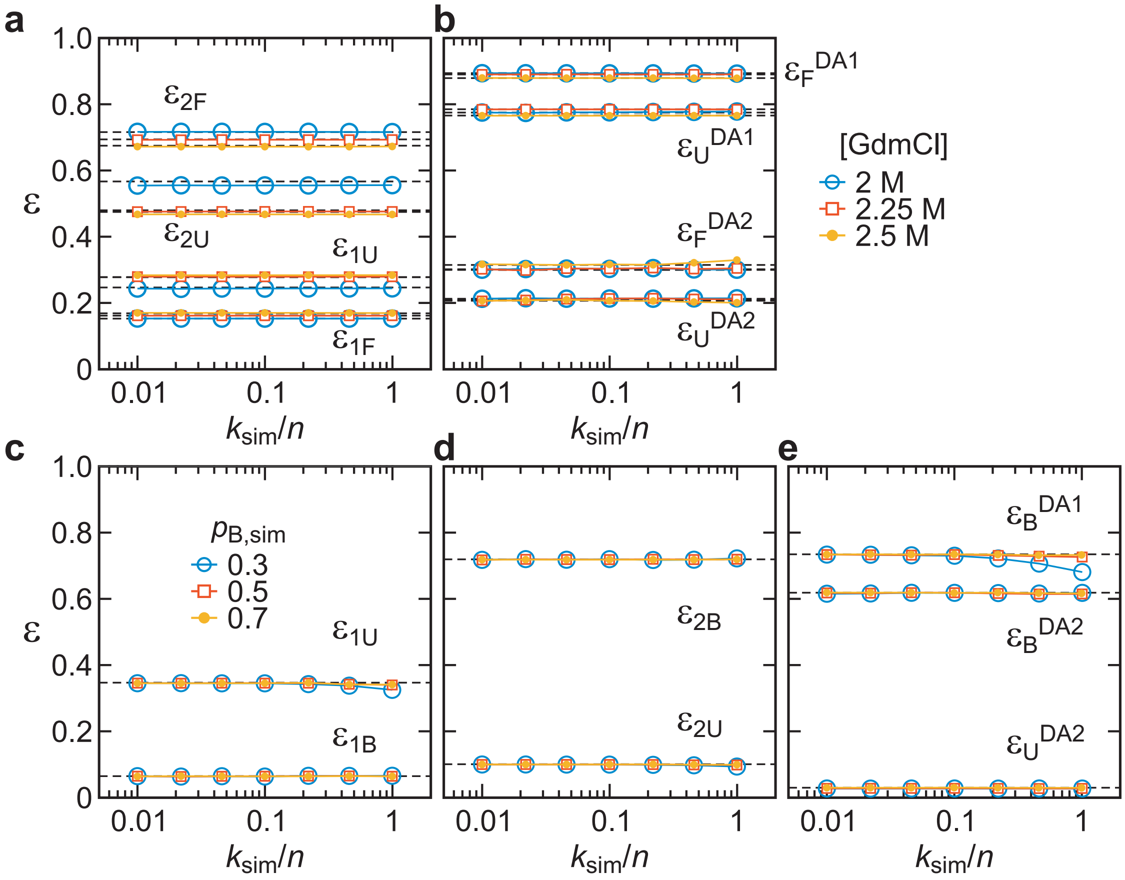

Supplementary Fig. 10. Fractions of the acceptor photon count rates extracted from the simulated photon trajectories using a two-state model with various relaxation rates ($k_{sim} = k_F + k_U$ for α3D folding and $k_{sim} = k_B + k_U$ for TAD/NCBD binding)) and three folded (p_F for α3D) or bound (p_B for TAD/NCBD) populations (see Fig. 6). n is the average photon count rate of the three-color segments, which are ~ 80 ms^{-1} for α3D folding and 65 ms^{-1} for TAD/NCBD binding. Horizontal dashed lines indicate the values in simulation. **a, b** α3D folding. **a** Three-color acceptor fractions ε_1 and ε_2. **b** Acceptor fractions for two-color parts of the trajectories: A1 fraction of the folded and unfolded states from DA1 segments ε_F^{DA1} and ε_U^{DA1} and A2 fraction of the folded and unfolded states from DA2 segments ε_F^{DA2} and ε_U^{DA2}. **c - e** TAD/NCBD binding. **c** Three-color A1 fraction ε_1. **d** Three-color A2 fraction ε_2. **e** Acceptor fractions for two-color parts of the trajectories: A1 fraction of the bound state from DA1 ε_B^{DA1}, and A2 fraction of the bound and unbound states from DA2 ε_B^{DA2} and ε_U^{DA2}. Source data are provided as a Source Data file.

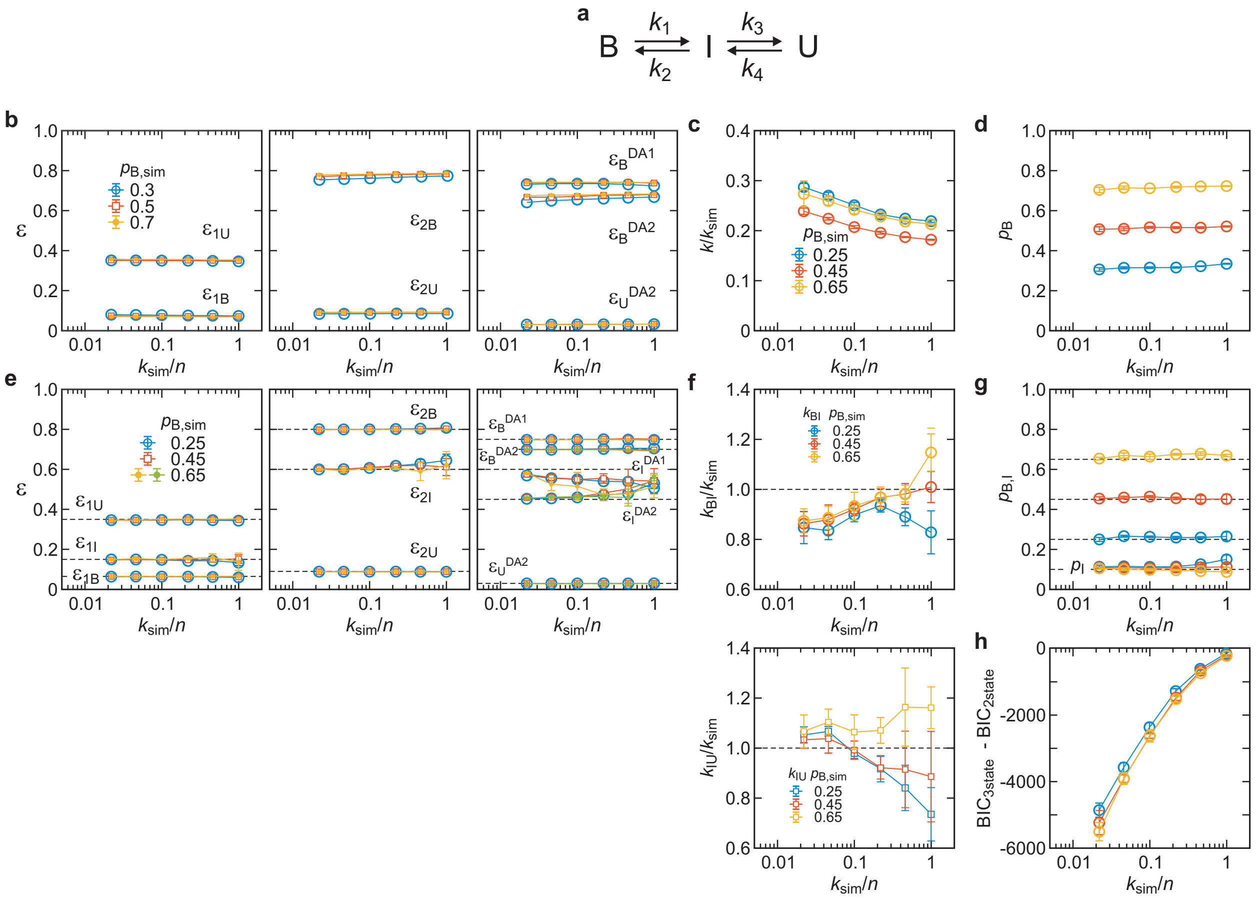

Supplementary Fig. 11. Three-state model binding simulation. Photon trajectories of TAD/NCBD binding at 0 mM NaCl were simulated by recoloring experimental photon trajectories using the three-state linear model and analyzed using two-state and three-state models for comparison. The relaxation rate is $k_{sim} = k_1 + k_2 = k_3 + k_4$, n is the average photon count rate of the three-color segments, which is 65 ms^{-1}. The fraction of the bound state p_B = 0.25, 0.45, and 0.65 in three simulation sets and the fraction of the intermediate state p_I = 0.1 for all simulations. Horizontal dashed lines indicate the values in simulation. **a** Three-state linear model. B, I, and U stand for the bound, intermediate, and unbound states, respectively. Blinking of A1 and A2 are also included in the simulation. **b – d** Maximum likelihood analysis using the two-state model. **b** Three-color A1 fraction ε_1 (left), three-color A2 fraction ε_2 (middle), and acceptor fractions for two-color parts of the trajectories: A1 fraction of the bound state from DA1 ε_B^{DA1}, and A2 fraction of the bound and unbound states from DA2 ε_B^{DA2} and ε_U^{DA2} (right). **c** Determination of the relaxation rate (k). **d** Determination of the fraction of the bound (p_B) state. **e – g** Maximum likelihood analysis using the three-state model. **e** Three-color A1 fraction ε_1 (left), three-color A2 fraction ε_2 (middle), and acceptor fractions for two-color parts of the trajectories (right). For the two-color acceptor fraction parameters with p_B = 0.65, different colors are used for the acceptor fractions of DA1 (orange dot) and DA2 (green dot) for the clear separation of the intermediate state acceptor fraction values. Among the acceptor fractions, the two-color acceptor fractions of the intermediate state, ε_I^{DA1} and ε_I^{DA2}, are the least accurate parameters. **f** Determination of the relaxation rates. $k_{BI} = k_1 + k_2$ (upper panel) and $k_{IU} = k_3 + k_4$ (lower panel). $k_{BI} = k_{IU}$ in the simulation. **g** Determination of the fraction of the bound (p_B) and intermediate (p_I) state. **h**

Difference of the Bayesian information criterion (BIC) values between the three-state and two-state model analyses. BIC = -2 ln L + n_p ln N_p, where L is the likelihood, n_p is the number of fitting parameters, and N_p is the number of photons analyzed. Error bars are standard deviations of the parameters obtained from five recolored data set. Source data are provided as a Source Data file.

Supplementary Table 1. Acceptor Fractions, FRET efficiencies, and kinetics parameters of α_3D folding.[a]

	8-state			2-state		
[GdmCl] (M)	2	2.25	2.5	2	2.25	2.5
ε_{1F}	0.153 (0.002)	0.162 (0.001)	0.169 (0.001)	0.156 (0.002)	0.162 (0.001)	0.170 (0.001)
ε_{1U}	0.247 (0.004)	0.278 (0.001)	0.278 (0.001)	0.249 (0.004)	0.280 (0.001)	0.283 (0.001)
ε_{2F}	0.716 (0.002)	0.694 (0.001)	0.675 (0.001)	0.712 (0.002)	0.693 (0.001)	0.672 (0.001)
ε_{2U}	0.567 (0.006)	0.480 (0.001)	0.475 (0.001)	0.546 (0.006)	0.476 (0.001)	0.468 (0.001)
ε_{F}^{DA1}	0.894 (0.001)	0.890 (0.001)	0.879 (0.001)	0.879 (0.001)	0.892 (0.002)	0.879 (0.001)
ε_{U}^{DA1}	0.775 (0.002)	0.785 (0.002)	0.766 (0.001)	0.764 (0.002)	0.782 (0.002)	0.764 (0.001)
ε_{F}^{DA2}	0.300 (0.039)	0.302 (0.012)	0.315 (0.010)	0.296 (0.002)	0.302 (0.005)	0.319 (0.003)
ε_{U}^{DA2}	0.213 (0.044)	0.207 (0.013)	0.209 (0.012)	0.211 (0.002)	0.206 (0.006)	0.210 (0.003)
k (ms^{-1})	1.16 (0.07)	1.05 (0.03)	0.84 (0.02)	1.05 (0.07)	1.09 (0.02)	0.91 (0.02)
p_F	0.64 (0.01)	0.47 (0.07)	0.29 (0.01)	0.68 (0.01)	0.47 (0.01)	0.30 (0.01)
k_{b1} (ms^{-1})	5.8 (1.1)	8.2 (0.5)	8.2 (0.6)			
p_{b1}[b]	0.997 (0.002)	0.998 (0.001)	0.991 (0.001)			
k_{b2} (ms^{-1})	1.3 (0.5)	1.5 (0.3)	1.1 (0.3)			
p_{b2}[b]	0.992 (0.001)	0.995 (0.001)	0.984 (0.001)			
BIC[c]	833976	2339912	2914394			
ΔBIC (3 state – 2 state)	-357	-281	-1387			
E_{1F}^{3c}	0.847 (0.010)	0.828 (0.004)	0.811 (0.004)	0.845 (0.009)	0.827 (0.004)	0.807 (0.004)
E_{1U}^{3c}	0.745 (0.013)	0.666 (0.003)	0.659 (0.002)	0.734 (0.013)	0.663 (0.002)	0.654 (0.002)
E_{12F}^{3c}	0.857 (0.010)	0.844 (0.004)	0.830 (0.004)	0.854 (0.009)	0.843 (0.004)	0.828 (0.004)
E_{12U}^{3c}	0.712 (0.013)	0.623 (0.003)	0.617 (0.002)	0.703 (0.013)	0.618 (0.002)	0.605 (0.002)
E_{2F}^{2c}	0.290 (0.038)	0.292 (0.011)	0.305 (0.010)	0.285 (0.002)	0.292 (0.002)	0.309 (0.002)
E_{2U}^{2c}	0.199 (0.041)	0.193 (0.012)	0.195 (0.011)	0.197 (0.002)	0.192 (0.001)	0.196 (0.001)

[a] Errors in parentheses are standard deviations calculated from the curvature at the maximum of the likelihood function. Errors of the corrected FRET efficiencies were estimated from error propagation in equations (7) – (9) by assuming that the errors of the acceptor fractions are independent as an approximation.

[b] p_{b1} and p_{b2} correspond to the values at the reference photon count rate of 100 ms^{-1}.

[c] BIC = -2 ln L + n_p ln N_p, where L is the likelihood, n_p is the number of fitting parameters, and N_p is the number of photons analyzed.

Supplementary Table 2. Acceptor Fractions, FRET efficiencies, and kinetics parameters of TAD/NCBD binding.[a]

	8-state			3-state		
[NaCl] (mM)	0	10	30	0	10	30
[NCBD] (nM)	25	100	200	25	100	200
ε_{1B}	0.065 (0.001)	0.058 (0.002)	0.062 (0.001)	0.072 (0.001)	0.066 (0.001)	0.067 (0.001)
ε_{1U}	0.347 (0.001)	0.351 (0.001)	0.352 (0.001)	0.343 (0.001)	0.342 (0.001)	0.343 (0.001)
ε_{2B}	0.719 (0.002)	0.747 (0.003)	0.762 (0.002)	0.708 (0.001)	0.725 (0.002)	0.749 (0.002)
ε_{2U}	0.101 (0.000)	0.129 (0.001)	0.145 (0.000)	0.101 (0.000)	0.129 (0.000)	0.143 (0.000)
ε_{B}^{DA1}	0.735 (0.001)	0.748 (0.002)	0.723 (0.002)	0.733 (0.001)	0.741 (0.002)	0.714 (0.002)
$\varepsilon_{U}^{DA1\,b}$	0.448	0.480	0.497	0.444	0.471	0.486
ε_{B}^{DA2}	0.619 (0.004)	0.586 (0.005)	0.617 (0.004)	0.602 (0.003)	0.571 (0.004)	0.603 (0.003)
ε_{U}^{DA2}	0.030 (0.000)	0.057 (0.000)	0.075 (0.000)	0.030 (0.000)	0.057 (0.000)	0.075 (0.000)
φ	0.36 (0.01)	0.37 (0.01)	0.28 (0.01)	0.35 (0.01)	0.35 (0.01)	0.25 (0.01)
$k'_{b2} + k'_{d2}$ (ms^{-1})	0.08 (0.01)	0.10 (0.02)	0.19 (0.03)	0.08 (0.01)	0.09 (0.01)	0.22 (0.03)
φ'	0.24 (0.03)	0.25 (0.05)	0.14 (0.04)	0.26 (0.04)	0.27 (0.05)	0.17 (0.03)
k (ms^{-1})^{c}	0.39 (0.01)	0.73 (0.02)	1.68 (0.04)	0.42 (0.01)	0.82 (0.02)	1.87 (0.04)
p_B	0.36 (0.01)	0.34 (0.01)	0.222 (0.005)	0.35 (0.01)	0.34 (0.01)	0.24 (0.01)
k_{b1} (ms^{-1})	7.5 (0.5)	19 (4)	12 (1)			
$p_{b1}{}^{d}$	0.985 (0.001)	0.969 (0.003)	0.977 (0.001)			
k_{b2} (ms^{-1})	55 (7)	59 (14)	19 (3)			
$p_{b2}{}^{d}$	0.978 (0.002)	0.972 (0.004)	0.958 (0.004)			
k_a (10^{9}M^{-1}s^{-1})	2.04 (0.08)	0.91 (0.03)	0.52 (0.02)			
k_d (ms^{-1})	0.25 (0.01)	0.48 (0.02)	1.30 (0.03)			
K_d (μM)	0.123 (0.005)	0.52 (0.02)	2.48 (0.07)			
BIC^{e}	2124867	1427593	3243679			
ΔBIC (3 state – 2 state)	-2230	-1223	-2148			
$E_{1B}^{3c\,f}$	0.727 (0.005)	0.787 (0.005)	0.803 (0.004)	0.729 (0.004)	0.767 (0.004)	0.793 (0.004)
E_{1U}^{2c}	0.411 (0.001)	0.433 (0.002)	0.439 (0.001)	0.406 (0.001)	0.423 (0.001)	0.427 (0.001)
E_{12B}^{3c}	0.897 (0.004)	0.928 (0.004)	0.920 (0.004)	0.886 (0.003)	0.911 (0.003)	0.911 (0.003)

E_{12U}^{3c}	0.015 (0.002)	0.026 (0.002)	0.030 (0.002)	0.018 (0.001)	0.034 (0.001)	0.034 (0.001)
E_{2B}^{2c}	0.744 (0.003)	0.706 (0.004)	0.728 (0.003)	0.730 (0.003)	0.691 (0.003)	0.715 (0.003)
E_{2U}^{2c}	-0.003 (0.001)	-0.004 (0.001)	0.002 (0.001)	-0.003 (0.002)	-0.004 (0.002)	0.002 (0.002)
E_{2B}^{3c} [g]	0.739 (0.004)	0.764 (0.006)	0.813 (0.004)	0.726 (0.004)	0.736 (0.005)	0.802 (0.003)
E_{2U}^{2c}	0.003 (0.004)	0.007 (0.005)	0.011 (0.004)	0.004 (0.003)	0.010 (0.004)	0.012 (0.003)
E_{12B}^{3c}	0.900 (0.001)	0.909 (0.001)	0.875 (0.002)	0.888 (0.001)	0.895 (0.001)	0.861 (0.002)
E_{12U}^{3c}	0.007 (0.003)	0.014 (0.005)	0.021 (0.003)	0.009 (0.003)	0.019 (0.004)	0.023 (0.003)
E_{1B}^{2c}	0.734 (0.001)	0.740 (0.002)	0.707 (0.002)	0.732 (0.001)	0.733 (0.001)	0.696 (0.002)
E_{1U}^{2c}	0.409 (0.001)	0.430 (0.001)	0.436 (0.001)	0.403 (0.001)	0.418 (0.001)	0.424 (0.001)

[a] Errors are standard deviations calculated from the curvature at the maximum of the likelihood function. Errors of the corrected FRET efficiencies were estimated from error propagation in equations (7) – (9) by assuming that the errors of the acceptor fractions are independent as an approximation.

[b] $\varepsilon_{U}^{DA1} = \varepsilon_{1U} + \varepsilon_{2U}$.

[c] $k = k_B + k_U$, where k_B is the apparent binding rate and k_U is the dissociation rate. The association rate coefficient in Fig. 5a is obtained by $k_a = k_B$/[NCBD] and the dissociation rate coefficient $k_d = k_U$.

[d] p_{b1} and p_{b2} correspond to the values at the reference photon count rate of 100 ms^{-1}.

[e] BIC = -2 ln L + n_p ln N_p, where L is the likelihood, n_p is the number of fitting parameters, and N_p is the number of photons analyzed.

[f] Three-color FRET efficiencies, E_{1B}^{3c}, E_{12B}^{3c}, and E_{12U}^{3c} in this block were obtained using E_{2B}^{2c} and E_{2U}^{2c}.

[g] Three-color FRET efficiencies, E_{2B}^{3c}, E_{12B}^{3c}, and E_{12U}^{3c} in this block were obtained using E_{1B}^{2c} and E_{1U}^{2c}.

Supplementary Table 3. Comparison of 8-state maximum likelihood parameters of partial set of TAD/NCBD binding data.[a]

[NaCl] (mM)	0		10		30	
Number of molecules	70	70	88	52	70	106
Number of segments	111 (3c/DA1)<br>72 (DA2)	112 (3c/DA1)<br>89 (DA2)	102 (3c/DA1)<br>49 (DA2)	63 (3c/DA1)<br>35 (DA2)	107 (3c/DA1)<br>110 (DA2)	177 (3c/DA1)<br>142 (DA2)
ε_{1B}	0.067 (0.001)	0.066 (0.001)	0.056 (0.003)	0.057 (0.004)	0.062 (0.002)	0.060 (0.002)
ε_{1U}	0.344 (0.001)	0.351 (0.001)	0.351 (0.001)	0.350 (0.002)	0.358 (0.001)	0.348 (0.001)
ε_{2B}	0.709 (0.003)	0.724 (0.002)	0.751 (0.004)	0.742 (0.005)	0.765 (0.004)	0.764 (0.003)
ε_{2U}	0.100 (0.000)	0.102 (0.001)	0.126 (0.001)	0.133 (0.001)	0.142 (0.001)	0.146 (0.001)
ε_{B}^{DA1}	0.738 (0.002)	0.733 (0.002)	0.749 (0.003)	0.744 (0.003)	0.721 (0.004)	0.725 (0.003)
ε_{B}^{DA2}	0.623 (0.006)	0.613 (0.005)	0.598 (0.006)	0.564 (0.009)	0.628 (0.005)	0.611 (0.006)
ε_{U}^{DA2}	0.035 (0.000)	0.026 (0.000)	0.056 (0.001)	0.059 (0.001)	0.072 (0.000)	0.079 (0.000)
φ	0.41 (0.02)	0.32 (0.02)	0.36 (0.02)	0.38 (0.02)	0.29 (0.01)	0.27 (0.01)
$k'_{b2} + k'_{d2}$ (ms^{-1})	0.07 (0.01)	0.07 (0.01)	0.13 (0.02)	0.07 (0.02)	0.14 (0.05)	0.24 (0.04)
φ'	0.20 (0.05)	0.12 (0.03)	0.23 (0.05)	0.28 (0.10)	0.09 (0.08)	0.16 (0.04)
k (ms^{-1})[b]	0.37 (0.02)	0.43 (0.02)	0.75 (0.03)	0.70 (0.03)	1.68 (0.06)	1.67 (0.05)
p_B	0.31 (0.01)	0.40 (0.01)	0.36 (0.01)	0.31 (0.01)	0.21 (0.01)	0.23 (0.01)
k_{b1} (ms^{-1})	6.6 (0.8)	7.9 (0.7)	22 (5)	13 (4)	11 (1)	13 (1)
p_{b1}[c]	0.986 (0.001)	0.985 (0.001)	0.967 (0.003)	0.973 (0.004)	0.979 (0.002)	0.976 (0.002)
k_{b2} (ms^{-1})	18 (4)	47 (6)	61 (18)	121 (81)	14 (2)	46 (12)
p_{b2}[c]	0.989 (0.003)	0.973 (0.003)	0.967 (0.005)	0.976 (0.008)	0.926 (0.009)	0.971 (0.005)

[a] Errors are standard deviations calculated from the curvature at the maximum of the likelihood function.

[b] $k = k_B + k_U$, where k_B is the apparent binding rate and k_U is the dissociation rate.

[c] p_{b1} and p_{b2} correspond to the values at the reference photon count rate of 100 ms^{-1}.

Supplementary Table 4. Relationship between three-color parameters in the presence of acceptor blinking and two-color or pre-determined parameters.

Acceptor dark state	3-color parameter	2-color parameter[a] DA1	2-color parameter[a] DA2	Pre-determined parameter[b]
A2 dark	$\varepsilon_{1S\mathrm{bd}}$	$\varepsilon_S^{\mathrm{DA1}}(1-\varepsilon_{\mathrm{d12}})$		
	$\varepsilon_{2S\mathrm{bd}}$	$\varepsilon_S^{\mathrm{DA1}}\varepsilon_{\mathrm{d12}}$		
A1 dark	$\varepsilon_{1S\mathrm{db}}$		$(1-\varepsilon_S^{\mathrm{DA2}})\varepsilon_{\mathrm{d1}}$	
	$\varepsilon_{2S\mathrm{db}}$		$\varepsilon_S^{\mathrm{DA2}}$	
A1, A2 dark	$\varepsilon_{1S\mathrm{dd}}$			$\varepsilon_{\mathrm{d1}}$
	$\varepsilon_{2S\mathrm{dd}}$			$\varepsilon_{\mathrm{d2}}$

[a] $\varepsilon_{\mathrm{d12}}$ is the fraction of A1 photons detected in the A2 channel, which can be determined from DA1 segments. Subscript S = F, U for folding and S = B, U for binding data.

[b] $\varepsilon_{\mathrm{d1}}$ ($\varepsilon_{\mathrm{d2}}$) is the fraction of photons detected in A1 (A2) channel after donor excitation when A1 and A2 are in the dark state, which can be pre-determined from the donor-only segments. $\varepsilon_{\mathrm{d1}} = n_{\mathrm{A1}}^0/(n_{\mathrm{D}}^0+n_{\mathrm{A1}}^0+n_{\mathrm{A2}}^0)$ and $\varepsilon_{\mathrm{d2}} = n_{\mathrm{A2}}^0/(n_{\mathrm{D}}^0+n_{\mathrm{A1}}^0+n_{\mathrm{A2}}^0)$, where n_{D}^0, n_{A1}^0, and n_{A2}^0 are the count rates in D, A1 and A2 channels, respectively, of the donor-only segment. In the binding experiment, $\varepsilon_{\mathrm{d2}} = \varepsilon_{\mathrm{U}}^{\mathrm{DA2}}$, which is determined from the maximum likelihood analysis of DA2 segments. Note that $\varepsilon_{\mathrm{d1}}$, $\varepsilon_{\mathrm{d2}}$, and $\varepsilon_{\mathrm{d12}}$ are not corrected for background photons, and therefore, are different from the leak values l_1, l_2, and l_{12} in equations (18) and (19).
