# Supplementary material for: Fast three-color single-molecule FRET using statistical inference
Source: Nat Commun. 2020 Jul 3;11:3336. doi: 10.1038/s41467-020-17149-w (PMC7335206; doi:10.1038/s41467-020-17149-w)
Supplement: Supplementary file 3 — Reporting Summary [file 41467_2020_17149_MOESM3_ESM.pdf]

nature research

Corresponding author(s): Hoi Sung Chung

Last updated by author(s): Jun 4, 2020

Reporting Summary

Nature Research wishes to improve the reproducibility of the work that we publish. This form provides structure for consistency and transparency in reporting. For further information on Nature Research policies, see Authors & Referees and the Editorial Policy Checklist.

Statistics

For all statistical analyses, confirm that the following items are present in the figure legend, table legend, main text, or Methods section.

n/a	Confirmed	
☒	☐	The exact sample size (*n*) for each experimental group/condition, given as a discrete number and unit of measurement
☒	☐	A statement on whether measurements were taken from distinct samples or whether the same sample was measured repeatedly
☒	☐	The statistical test(s) used AND whether they are one- or two-sided<br>*Only common tests should be described solely by name; describe more complex techniques in the Methods section.*
☒	☐	A description of all covariates tested
☒	☐	A description of any assumptions or corrections, such as tests of normality and adjustment for multiple comparisons
☐	☒	A full description of the statistical parameters including central tendency (e.g. means) or other basic estimates (e.g. regression coefficient) AND variation (e.g. standard deviation) or associated estimates of uncertainty (e.g. confidence intervals)
☒	☐	For null hypothesis testing, the test statistic (e.g. *F*, *t*, *r*) with confidence intervals, effect sizes, degrees of freedom and *P* value noted<br>*Give P values as exact values whenever suitable.*
☒	☐	For Bayesian analysis, information on the choice of priors and Markov chain Monte Carlo settings
☒	☐	For hierarchical and complex designs, identification of the appropriate level for tests and full reporting of outcomes
☒	☐	Estimates of effect sizes (e.g. Cohen's *d*, Pearson's *r*), indicating how they were calculated

Our web collection on statistics for biologists contains articles on many of the points above.

Software and code

Policy information about availability of computer code

Data collection	Symphotime v5 (Picoquant), Matlab
Data analysis	Matlab and custom codes, which are available at https://github.com/hoisunglab/FRET_3colorCW

For manuscripts utilizing custom algorithms or software that are central to the research but not yet described in published literature, software must be made available to editors/reviewers. We strongly encourage code deposition in a community repository (e.g. GitHub). See the Nature Research guidelines for submitting code & software for further information.

Data

Policy information about availability of data

All manuscripts must include a data availability statement. This statement should provide the following information, where applicable:

- Accession codes, unique identifiers, or web links for publicly available datasets
- A list of figures that have associated raw data
- A description of any restrictions on data availability

All data supporting the main conclusion are included in the manuscript. Extra data are available from the corresponding author upon reasonable request. The source data underlying Figs. 4, 5, and 6, and Supplementary Figs. 2b, 2c, 6e, 7 – 10, and 11b – 11h are provided as a Source Data file.

Field-specific reporting

Please select the one below that is the best fit for your research. If you are not sure, read the appropriate sections before making your selection.

☒ Life sciences ☐ Behavioural & social sciences ☐ Ecological, evolutionary & environmental sciences

October 2018

Life sciences study design

All studies must disclose on these points even when the disclosure is negative.

Sample size	We have included the number of molecules analyzed in the caption of Fig. 3. The number of molecules are sufficient to determine parameters accurately as demonstrated in the simulations in Fig. 6.
Data exclusions	We observe the spectral shift of the donor fluorophore (Alexa 488) occasionally, which changes the FRET efficiencies. Therefore, the trajectories affected by the spectral shift were excluded from the analysis. The spectral shift can be identified by increased donor leak into the acceptor channel as described in several papers including Chung et al. PNAS vol. 106, 11837 (2009).
Replication	At each experimental condition (different NaCl or chemical denaturant concentration), the data were collected in a single experiment. However, a large number of molecules and photons are analyzed and no outlier from the experimental trend was identified. The measured kinetic parameters are also consistent with the previous results for both systems (J Phys Chem B, vol. 122, 11702 (2018), Nat Commun, v9, 4707(2018)), which ensures the reproducibility of the measurements. In addition, for the binding experiment, the reproducibility was tested by analyzing the data after splitting into two sets, which results in similar maximum likelihood parameters (Supplementary Table 3).
Randomization	There should be no systematic bias among different individual molecules. No randomization is required.
Blinding	Blinding is not applicable in this study of protein molecules.

Reporting for specific materials, systems and methods

We require information from authors about some types of materials, experimental systems and methods used in many studies. Here, indicate whether each material, system or method listed is relevant to your study. If you are not sure if a list item applies to your research, read the appropriate section before selecting a response.

Materials & experimental systems

n/a	Involved in the study
☒	☐ Antibodies
☒	☐ Eukaryotic cell lines
☒	☐ Palaeontology
☒	☐ Animals and other organisms
☒	☐ Human research participants
☒	☐ Clinical data

Methods

n/a	Involved in the study
☒	☐ ChIP-seq
☒	☐ Flow cytometry
☒	☐ MRI-based neuroimaging
